# Factors Affecting Village Apparatus, Integrated Service Post and Early Childhood Education in Stunting Prevention

**DOI:** 10.4314/ejhs.v33i2.8

**Published:** 2023-03

**Authors:** Sri Mulyani, Soetrisno, Tri Rejeki Andayani, Grhasta Dian Perestroika

**Affiliations:** 1 Vocation School of Universitas Sebelas Maret, Surakarta, Indonesia; 2 Postgraduate Program of Universitas Sebelas Maret, Surakarta, Indonesia; 3 Faculty of Medicine Universitas Sebelas Maret, Surakarta, Indonesia; 4 Faculty of Psychology Universitas Sebelas Maret, Surakarta, Indonesia

**Keywords:** Stunting, Prevention, Village, Posyandu, PAUD

## Abstract

**Background:**

The study aimed to identify the achievement of stunting prevention based on the contribution of the Village Apparatus, Integrated Health Services Post and Early Childhood Education. Moreover, it determined the effect of the personnel factors of each agency on the achievement of stunting prevention.

**Methods:**

A cross-sectional study was conducted on 175 respondents in Surakarta residences covering Klaten, Boyolali, Karanganyar, Surakarta, Sukoharjo, and Sragen districts from August to October 2021. The researcher conducted a line plot to describe the score of stunting prevention efforts through SINAR APD in planning, funding, implementation, and monitoring. Linear regression and One Way Anova were analyzed using SPSS to determine the effect of the personnel factors.

**Results:**

Personnel factors had a significant role in the achievement of funding for Stunting Prevention. The data of stunting showed knowledge (F=3.3; P<0.05), attitudes towards authoritative parenting (F=5.6; P<0.05), and attitudes towards permissive parenting (F=6.1; P<0.05).

**Conclusions:**

The main problem was the lack of achievement in the funding aspect. The researcher recommended further research to increase knowledge on stunting for village apparatus, Integrated Health Services Post and Early Childhood Education. Understanding good parenting patterns can change mindsets and attitudes to avoid applying parenting methods that are too authoritarian or permissive.

## Introduction

Stunting is a condition in which a child's height is below age. Stunting is a form of growth failure (growth faltering) due to the accumulation of nutritional inadequacy that lasts for a long time, starting from pregnancy until 24 months (1,2). The indicator used to identify stunted toddlers is based on the height for age index according to WHO child standards. Growth standard with stunting criteria if the z score < -2 Standard Deviation (SD) (3). The impact of stunting is an increased risk of morbidity and mortality and suboptimal brain development, so the development is delayed, and mental growth is stunted (4,5).

Stunting is a problem that must be serious because children are the future generation and reflection of Indonesia's future. The stunting of children under five is a consequence of several factors that often occur: poverty, social and culture, food insecurity, and public access to health services (3,6). Many factors cause stunting in children under five. The direct cause is a lack of food intake and infectious diseases. Other factors are the mother's lack of knowledge, wrong parenting, poor hygiene and sanitation, and low health services (7,8).

The incidence of stunting is indirectly influenced by socioeconomic factors, such as education level, family income, and food availability (9). Food availability is the family's ability to meet sufficient food needs in quantity, quality, and safety. Poor conditions can also be seen from the difficulty of all access, including access to information(10). It is where the role of the Village/Kelurahan apparatus is needed to identify family backgrounds with middle to lower economic levels with various characters in their area. With the existing village funds, village apparatus can carry out economic empowerment through entrepreneurship education to be independent.

Maternal health and nutrition conditions before and during pregnancy and after delivery affect fetal growth and the risk of stunting. These risky conditions include the failure of exclusive breastfeeding and the early weaning process, limited health services, including ANC services - Ante Natal Care (health services for mothers during pregnancy), Post Natal Care, and pregnant women who have not consumed supplements. adequate iron (11). ANC is a health service provided by health workers for mothers during pregnancy. The fact is that there are still many mothers who consider pregnancy as normal, natural, and natural, so they don't feel the need to check their pregnancy regularly with health services. This poor health behavior causes the risk factors that may be experienced by mothers to be undetected from an early age (12). Posyandu (Integrated health center), as the spearhead in stunting prevention, needs to revitalize class activities for pregnant women for quality ANC, postnatal, and early learning services.

Parenting is a factor that is closely related to the growth and development of children under five years of age. Various aspects of parenting (parents) include feeding practices, psychosocial stimulation, hygiene/hygiene practices, environmental sanitation, and health services utilization. The growth and development of toddlers are irreversible (cannot be recovered), so children at the age of toddlers need quality parenting (13). The low quality of parenting causes the poor nutritional status of toddlers (14). *PAUD* institutions*-Pendidikan Anak Usia Dini* (Early Childhood Education), both non-formal, such as daycare centers and playgroups, and formal preschool, need to optimize their role and improve the capacity of their human resources to stimulate and see all aspects of child growth and development in the context of preventing stunting.

The government has to provide support. This support is manifested in a stunting prevention program by empowering and synergizing officials and officers from three relevant agencies: the apparatus village, *Posyandu-Pos Layanan Kesehatan Terpadu* (Integrated Health Services Post), and *PAUD*. Previous studies in the same series of studies have successfully validated the measurement model of the four aspects or dimensions of stunting prevention efforts: planning, funding, implementation, and monitoring. In the model, various individual factors of personnel from the three related agencies are added, including knowledge about stunting, attitudes toward parenting, and attitudes toward children's eating patterns. Path analysis (part of the SEM analysis for the components of the relationship between variables) shows that, generally, these factors do not have a significant effect. These findings were obtained by using individual personnel as the unit of analysis. Because the achievement in question is an effort and synergy between agencies, a more precise assessment should be carried out at the agency level. Thus, the purpose of this study is to identify the achievement of stunting prevention efforts based on each agency's contribution and to examine the influence of personnel factors from each agency on these achievements.

## Materials and Methods

This research received ethical approval from the ethics committee of the medical faculty of UNS No. 43/UN27.06.6.1/KEP/EC/21. A cross-sectional study was conducted on 175 respondents in Surakarta residences covering Klaten, Boyolali, Karanganyar, Surakarta, Sukoharjo, and Sragen districts from August to October 2021. Research subjects are Village Apparatus, *Posyandu-Pos Layanan Kesehatan Terpadu* (Integrated Health Services Post)*,* and *PAUD* institutions*-Pendidikan Anak Usia Dini* (Early Childhood Education).

The variables in this study included four aspects of stunting prevention efforts which included funding, planning, implementation, and monitoring through the synergy of village apparatus, *Posyandu* and *PAUD*, staff knowledge about stunting, dimensions of attitudes towards parenting, and attitudes towards eating patterns and knowledge of officers about stunting. The measurement of the variable was obtained by calculating the average score of all the indicators that measure it. The score of each variable for each agency is obtained by calculating the average variable score of all personnel in the relevant agency. It assessed all variables collectively and provides an advantage in reducing the subjectivity factor.

Two stages of analysis were carried out in this study. First, a line plot of the average score of stunting prevention efforts through SINAR APD was made in each aspect (planning, funding, implementation, monitoring) for each agency from the seven research locations. SINAR APD was an acronym of “*Sinergi aparat Desa, Posyandu dan PAUD*”, the name of program in bahasa Indonesia. SINAR APD was the model of empowering and synergizing officials and officers from three relevant agencies: Village Apparatus, *Posyandu* and *PAUD*. Based on the plot, the level of achievement of each location is classified. Second, a statistical test of the influence of personnel factors from each agency was carried out on the level of achievement of stunting prevention efforts. The test technique used was multiple linear regressions with a significance level of for the statistical test of 5%.

The results of linear regression analysis obtained interrelated variables (three parenting models), so it can be ascertained that it will cause covariance or even multi-collinearity. Therefore, the researchers conducted further analysis on the influence of personnel factors on the achievement of stunting prevention efforts through SINAR APD with one-way ANOVA and Post Hoc tests. The analysis was performed in SPSS for Windows.

## Results

**The mean score of stunting prevention efforts through SINAR APD**: The higher the mean score means the better the efforts of each agency. The synergy, the highest level of achievement, is obtained when the average score of the three agencies is high and homogeneous. The results show various levels of achievement of stunting prevention efforts through SINAR APD referring to the measurement instruments used in this study: very good, good, moderate, poor, and very poor.

**Figure 1:**
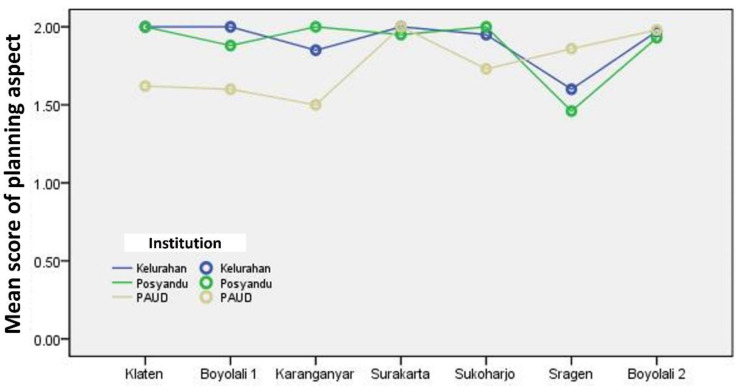
Line plot of the mean score of the Planning Aspect for Stunting Prevention Efforts through SINAR APD (Source: Output of SPSS for Windows, 2021)

Figure 1 shows that all line plots are in areas with an average score of 1.5 to 2. It shows that, in general, the planning aspects of stunting prevention efforts through SINAR APD from the seven research locations are good.

**Figure 2:**
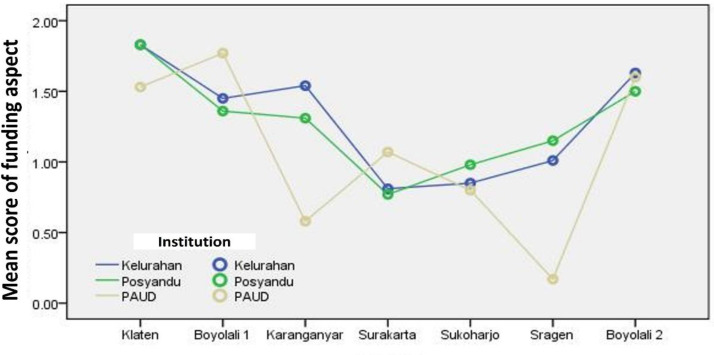
Line plot of the mean score of Funding Aspect for Stunting Prevention Efforts through SINAR APD (Source: Output of SPSS for Windows, 2021)

Figure 2 shows that the line plots are widely spread from the lowest mean score (0) to the highest mean score (2). More importantly, most points are below the average score of 1.5, and many are below the average score of 1. It shows the funding aspect in stunting prevention efforts through PPE SINAR from most research sites, including less. Compared to the planning aspect, there are very serious problems in the funding aspect. This condition is very likely to be a determinant of poor output because there is no dispute that funding is a major factor in the success or implementation of a program.

**Figure 3:**
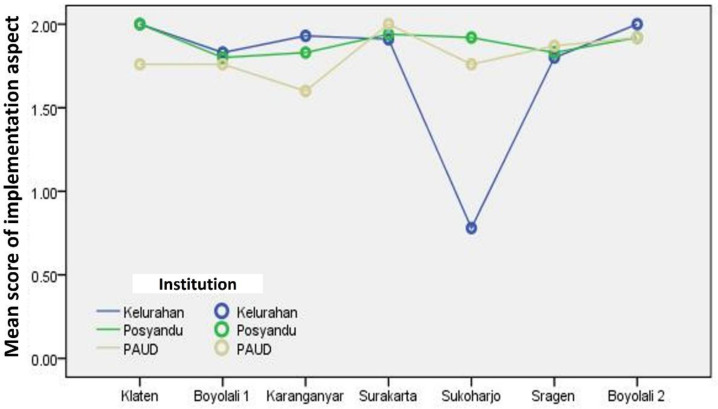
Line plot of the mean score of the Implementation Aspect for Stunting Prevention Efforts through SINAR APD (Source: Output of SPSS for Windows, 2021)

Figure 3 shows that the line plots are all in the area with an average score of 1.5 to 2. It shows that the implementation aspects of stunting prevention efforts through SINAR APD from the research location are good.

**Figure 4:**
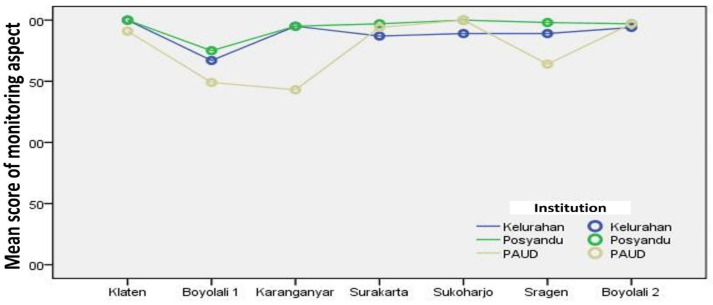
Line plot of the mean score of Monitoring Aspect for Stunting Prevention Efforts through SINAR APD (Source: Output of SPSS for Windows, 2021)

Figure 4 shows that the line plots are almost entirely in the area with an average score of 1.5 to 2. It shows that the monitoring aspect in stunting prevention efforts through SINAR APD from the seven research locations is generally quite good. The monitoring aspect can be said to be good among the four activity aspects.

**Effect of the personnel factors**: Table 1 shows the beta coefficient values and the statistical significance of the linear regression model, which states the influence of personnel factors on the level of achievement of stunting prevention efforts through PPE RAIN. Of the four aspects, the personnel factor significantly affects only the funding aspect. Personnel factors influencing the funding aspect are attitudes towards democratic parenting (β = 0.469; p = 0.046) and attitudes towards permissive parenting (β = -0.604; p = 0.005). The interpretation is that the more positive the attitude (supportive) towards democratic parenting and the more negative (unsupportive) attitude towards permissive parenting, the better the achievement of funding aspects in stunting prevention efforts through APD SINAR. As for aspects other than funding, no significant relationship was found with personnel factors.

**Table 1:** Analysis of the Influence of Personnel Factors on the Achievement of Stunting Prevention Efforts through SINAR APD

Personnel Factor	Planning	Funding		Implementation	Monitoring

	β^a^	β^a^	F^b^	β^a^	β^a^
Knowledge of stunting	-0,318	-0,190	3,264*	-0,258	0,026
Attitudes toward authoritative parenting	0,336	-0,048	5,583*	0,075	0,186
Attitudes toward democratic parenting	0,240	0,469*	0,960	-0,012	0,245
Attitude toward permissive parenting	-0,047	-0,604*	6,127*	-0,199	-0,104
Attitude to diet	0,421	0,292	0,904	0,099	0,350

Note: Score level, very good = 5, good = 4, moderate = 3, less = 2, very poor = 1

* p < 0.05

a Linear Regression Analysis

b One Way Anova

Source: output of SPSS for Windows, 2021

In the regression model, covariance or the relationship between factors is considered a component that reduces the partial effect of the influence of factors on the dependent variable. The concept applies mathematical calculation so that it cannot identify whether the relationship conceptually or factually does exist or is just a coincidence. In this study, personnel factors are conceptually unrelated because the response objects for certain cognitive or affective dimensions of the individual are different. The response object from the knowledge dimension is stunting (the problem under study), while the response object from the attitude dimension is parenting and eating patterns (a factor related to the problem under study, namely stunting). Attitudes towards eating patterns are distinguished from attitudes towards parenting because of their specificity, which only concerns how parents (mothers) provide food consumption (nutritional intake) for their children. Attitudes toward parenting are divided into three to assess individual tendencies or support for certain forms of parenting. The three forms of parenting are interrelated, so it will certainly cause covariance or even multi-collinearity (too strong a correlation between independent variables in linear regression models). A more in-depth analysis was carried out on the influence of personnel factors on the achievement of stunting prevention efforts through SINAR APD. The analysis is only carried out on the funding aspect, which shows serious problems and wide variations in the level of achievement.

With one-way ANOVA analysis, the relationship of each personnel factor with the level of cooperation is tested separately to ignore the possibility of a correlation between personnel factors. As a result, it was found that the level of stunting prevention efforts through SINAR APD related to factors about stunting, authoritative parenting attitudes, and attitudes towards permissive parenting. Further testing of analysis of variance (post hoc test) with Duncan's test approach showed that the research location was about higher education, had the highest score of knowledge about stunting and attitudes towards authoritative parenting, and had the lowest consent (Table 2).

**Table 2:** Post Hoc Test (Duncan's Test) Comparison of Personnel Factor Scores Based on the Achievement Level of Stunting Prevention Efforts through PPE RAINS on Funding Aspects

Achievement Level	Knowledge of stunting	Attitudes toward authoritative parenting	Attitude toward permissive parenting
Good	1,84^a^	1,53^a^	2,01^a^
Moderate	1,67^b^	2,12^b^	2,41^b^
Poor	1,68^b^	1,99^b^	2,59^b^
Very poor	1,75^ab^	2,19^b^	2,59^b^

## Discussion

The assessment of the achievement of stunting prevention efforts through the SINAR APD above was carried out with the criteria specifically determined for this study based on the format of the research instrument and the concept of synergy between agencies as the most important aspect of the success of the program. A comparative statistical test was conducted to compare the average scores of stunting prevention efforts between study sites to validate the assessment results. The results were consistent with the assessment interpretation based on the plots and criteria established specifically for this study. In planning, implementation, and monitoring, the average achievement scores of all locations are in the range of 1.5 and 2. As for the funding aspect, the average achievement scores of the seven research locations vary widely, some are above 1.5, but some are below 1. Statistically, the average achievement score between locations in the funding aspect is statistically different. The interpretation of this statistical test is that there are good achievements in planning, implementation, and monitoring. However, there is a problem of lack of stunting prevention efforts through SINAR APD in the funding aspect.

With a shortage in the funding aspect, it is possible to get results or outcomes (reducing the incidence of stunting) that are not good. Several personnel factors, such as knowledge about stunting and attitudes towards authoritative and permissive parenting, have been shown to affect the achievement of efforts in the funding aspect. Previous studies have proven the influence of village apparatus competence has a significant impact on village fund management accountability (15). Lack of knowledge about stunting and attitudes toward parenting are associated with the lack of competence in stunting resolution. Competent officers will produce good output, this is in line with the theory of human resources which states that resources must be improved in quality and competence to become strong (16). The finding in present study also in line with previous study which explains that the competence of human resources and fund management has an effect on financial quality (17).

There are several limitations to this study. As the survey was cross-sectional, no causal interpretations can be made. The survey used in this study relies mainly on self-reports questionnaire, some of which may be misreported due to respondent's competence reputation. To minimize the misreported in the questionnaire, we explain that their answers are confidential, only the analyzed data will be published.

Nevertheless, this study may contribute to determine the main problem of affecting factor of village apparatus, integrated service post and early childhood education in stunting prevention. It was the lack of achievement in the funding aspect. It is recommended to increase knowledge on stunting for village apparatus, Posyandu, and PAUD, as well as provide an understanding of good parenting patterns so that they can change mindsets and attitudes to avoid applying parenting methods that are too authoritarian or too permissive. Thus, it can increase personal competence which has an impact on improving financial management.
